# Clinical outcomes of a digital musculoskeletal women’s pelvic health program: an observational, longitudinal study with comparison group

**DOI:** 10.1186/s12905-024-03475-4

**Published:** 2025-01-11

**Authors:** Mindy Hong, Rachel Foster Kirk, Bijal Toprani, Cynthia Castro Sweet, Clare Pan, Jeffrey Krauss, Tamara Grisales

**Affiliations:** 1 Hinge Health, Inc, 455 Market Street, Suite 700, San Francisco, CA 94105 USA; 2 Department of Obstetrics & Gynecology, University of California Los Angeles, 200 Medical Plaza, Los Angeles, CA 90095 USA; 3 Teladoc Health Inc, 2 Manhattanville Rd, Purchase, NY 10577 USA

**Keywords:** Exercise therapy, Pelvic pain, Women’s health, Musculoskeletal pain, Telemedicine, Telerehabilitation

## Abstract

**Background:**

Chronic pelvic pain is a common yet undertreated condition that significantly impacts quality of life for women worldwide. Digital exercise therapy designed to target pelvic pain can improve symptomology while reducing time and cost-related barriers to in-person clinical care.

**Methods:**

This longitudinal, observational study of a digital women’s pelvic health program examined pelvic pain, anxiety, and depression at 4 and 12 weeks in female adults experiencing chronic pelvic pain. Intervention participants received a digital pelvic health program including personalized exercise therapy sessions, health education articles, and health coaching. A comparison group of nonparticipants received a series of education articles related to pelvic health. Data were collected at baseline, 4 and 12 weeks. Unadjusted and adjusted linear mixed effects models were conducted to model changes in clinical outcomes over time.

**Results:**

A total of 797 participants (intervention: 495, nonparticipants: 302) were included in the sample. Baseline mean (SD) age was 41.5 (11.7) years and mean pain was 45.7 (18.5) out of 100. Compared to baseline, the intervention group showed significantly more pain improvement at 4 and 12 weeks versus nonparticipants after adjusting for baseline factors. The intervention group’s pain scores decreased by 44.5% at 4 weeks and 53.6% at 12 weeks. The intervention group’s adjusted pain scores decreased from 42.0 (95% CI: [39.4, 44.7]) at baseline to 23.3 (95% CI: [20.5, 26.2]) at 4 weeks to 19.5 (95% CI: [16.7, 22.4]) at 12 weeks. In contrast, nonparticipants’ pain scores decreased by 21.6% at 4 weeks and 32.7% at 12 weeks. Nonparticipants’ adjusted pain scores decreased from 42.1 (95% CI: [38.4, 45.9]) at baseline to 33.0 (95% CI: [29.2, 36.8]) at 4 weeks to 28.3 (95% CI: [24.5, 32.2]) at 12 weeks. After adjustments, the probability of the intervention group screening for moderate or severe depression was significantly lower by 11.0% at 12 weeks versus nonparticipants. There were no significant differences in anxiety outcomes between groups at baseline, week 4, or week 12.

**Conclusions:**

A digital women’s pelvic health program may help reduce short-term pelvic pain and depression symptoms.

**Trial registration:**

The WIRB-Copernicus Group Institutional Review Board (registration number IRB20234932) approved this study on November 6, 2023.

**Supplementary Information:**

The online version contains supplementary material available at 10.1186/s12905-024-03475-4.

## Background

Chronic pelvic pain (CPP) impacts 26% of women worldwide and can have a significant negative impact on functioning and quality of life [1]. CPP is pain that has been present for at least 3 months, cyclic or non-cyclic, and may or may not be related to dysmenorrhea [2, 3]. Causes of CPP are multifaceted and not well understood; in 80% of the cases, CPP is not gynecologic and occurs due a combination of biopsychosocial factors [1]. Pain catastrophizing, poor general health, low quality of life, and sexual dysfunction are often comorbid in women with CPP [4–7]. Headaches occur in up to 60% of women in CPP, and backaches occur in up to 90% of women with CPP [8]. Women with CPP experience a wide range of physical and psychological challenges related to their condition that pose a serious risk to their wellbeing.

Treatment of CPP in women presents a substantial economic burden to the United States healthcare system, with estimates of annual healthcare costs at $2.8 billion dollars or $16,970 to $20,898 per woman per year [9]. Additionally, CPP results in an estimated annual cost of $15 billion dollars due to loss of productivity [10]. Due to the complex etiology of CPP, typical treatments are largely limited to symptom relief and focused on multidisciplinary management (i.e., analgesics, antidepressants, hormone treatment, cognitive therapy) [11]. However, these treatments are many times ineffective and often fail to sufficiently relieve pain in a large number of women [11]. Moreover, there is high variability in clinical practice guidelines for the diagnosis and management of CPP in women; many recommendations lack good quality evidence, which poses a large threat to the accessibility of safe and effective treatment for this condition [12]. The financial impact of CPP treatment, emphasis on symptom management, and lack of consistency in clinical guidelines highlight the necessity for CPP solutions that are cost effective, clinically validated, and focused on preventative strategies.

Pelvic floor physical therapy (PFPT) is an evidence-based therapy that targets the prevention and treatment of abdominal and pelvic functional disorders [13]. In recent years, telerehabilitation or digital therapy has become more common in delivering physical therapy care [14]. Given their ability to allow users to access services at all hours and locations, these approaches provide an opportunity for women to access treatment for pelvic pain, circumventing barriers related to receiving in-person therapy. Additionally, participants of PFPT programs have seen significant improvements in pelvic pain [15], with a recent systematic review demonstrating medium and large effect sizes in severity of urinary incontinence, pelvic floor muscle strength, and quality of life [16] .

While the evidence base for the impact of digital PFPT is expanding, research on the efficacy of digital PFPT for women with CPP is in a nascent stage, while more literature focuses on the impact of PFPT for urinary incontinence than on general CPP. To address this, this study aimed to evaluate the impact of a novel digital women’s pelvic health program on pelvic pain, anxiety, and depression at 4 and 12 weeks for women experiencing CPP.

## Methods

### Study design

A prospective, longitudinal, observational study comparing participants in a digital women’s pelvic health program (i.e., intervention) versus a nonparticipant comparison group at 4 and 12 weeks was conducted. The program lasted a total of 12 weeks, and participants were encouraged to engage in exercise therapy at least three times per week. Exercise therapy sessions were continuously available within the app to allow participants to engage at any time.

### Women’s pelvic health program

Employers offered the digital women’s pelvic health program (Hinge Health, Inc., San Francisco, CA) to employees and adult dependents as a health benefit. Recruitment was conducted through post and email. Members registered for the program by completing an online eligibility application questionnaire and accessed the app on their personal tablets or smartphones.

**Fig. 1:**
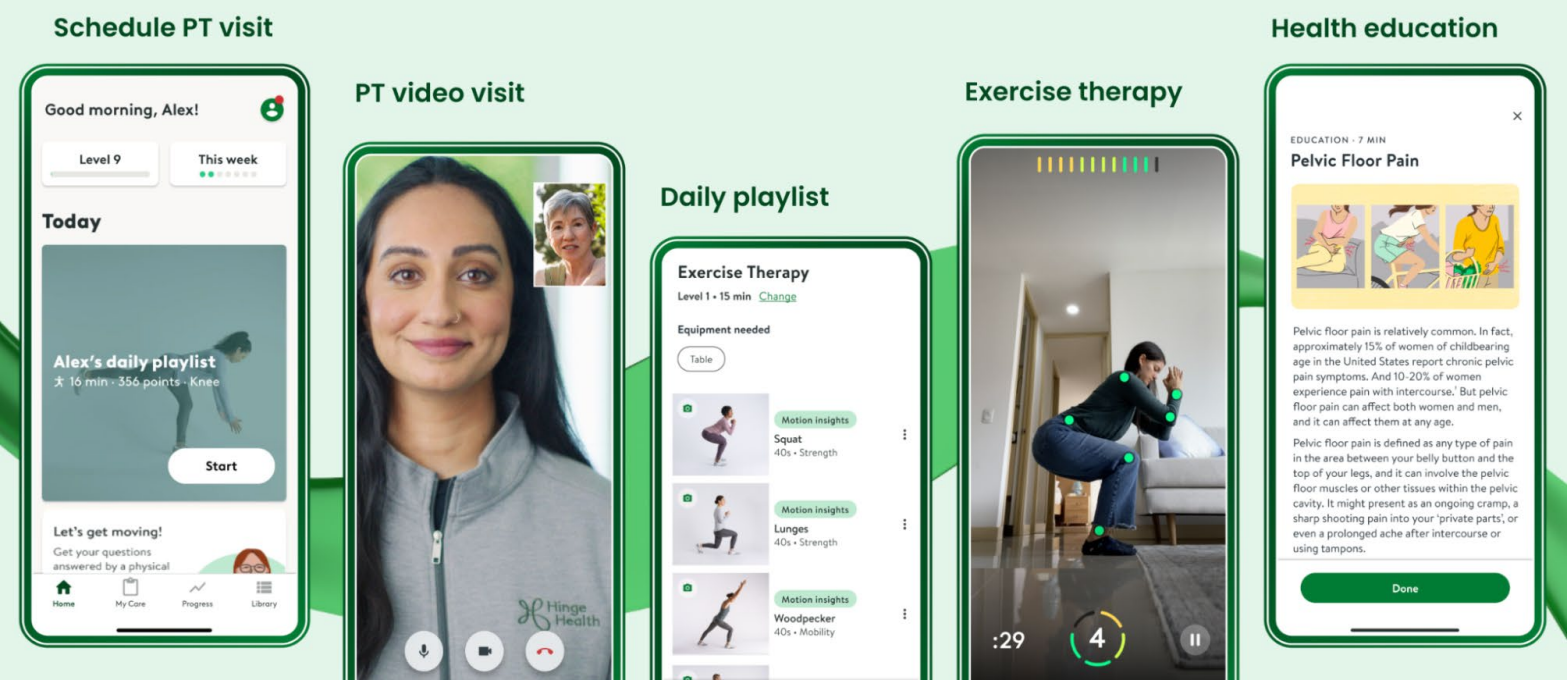
Digital women’s pelvic health program

Developed by physical therapists (PTs), the women’s pelvic health program aimed to help participants address their pelvic pain through access to a clinical care team consisting of Nationally Board Certified (NBC-HWC) health coaches and physical therapists trained in the assessment and treatment of pelvic floor conditions, pelvic floor exercise therapy sessions, and health education (Fig. 1). Each exercise session presented a set of exercises that target relaxing and controlling the pelvic floor muscles tailored to the participants’ preferences, goals, and symptoms. As participants advanced through the program, their exercises were adjusted by a PT in order to progress them towards their goals. Individual goals were determined between the participant and their PT, and ranged from goals based on physical function, frequency of engagement with the program, and lifestyle modification. Participants were encouraged to complete 3 or more 15–20 min exercise sessions per week, and worked with their care team to determine an optimal level of engagement that was feasible for them. Immediately after participants completed their exercise sessions, they received a new educational article about pelvic pain-related topics, such as pain neuroscience, treatment options, lifestyle practices, and relaxation techniques. Additionally, these education articles were made available within the app for viewing at any time. As an entirely digital program, participants could choose when and where to meet with PTs, complete exercise sessions, and read educational articles through the smartphone app. Participants could schedule a video visit with their care team or discuss changes to exercise therapy treatment plans at any time throughout the study.

### Study participants

Each week between November 2023 and February 2024, individuals meeting the inclusion and exclusion criteria based on information provided in the application were identified. Inclusion criteria were female, between the ages of 18–85, had an email account and a smartphone, experienced pelvic pain within the last 3 months, and had a Numeric Pain Rating Scale (NPRS) score $\:\ge\:$ 20 over the past 24 hours. Exclusion criteria were pregnancy, given birth within the last year, undergone pelvic surgery (i.e., hysterectomy or bladder sling placement) within the last 3 months, active pelvic infection (i.e., urinary tract infection or pelvic inflammatory disease), diagnosis of gastrointestinal or urinary disorders, history of opioid, alcohol, or drug abuse within the last year, and diagnosis of cognitive, behavioral, neurologic, or psychiatric disorders.

Intervention group participants and nonparticipants were recruited via different methods. Intervention group participants were recruited from members who had joined the digital women’s pelvic health program within one week prior to recruitment. Nonparticipants were recruited through an external recruitment firm.

### Outcomes

The primary outcome was pain improvement as measured by the Numeric Pain Rating Scale (NPRS [17]), based on the response to the question: “Over the past 24 hours, how bad was your pelvic pain?” with a score ranging from 0 (none) to 100 (worst imaginable).

A secondary outcome was risk for moderate or severe depression, which was measured by the 2-item Patient Health Questionnaire (PHQ-2) where those who screened positive (i.e., a score of 3 or higher) were determined to have risk for moderate or severe depression [18]. The last secondary outcome was risk for moderate or severe anxiety, as measured by the Generalized Anxiety Disorder 2-item questionnaire (GAD-2), where those who screened positive (i.e., score of 3 or higher) were determined to have risk for moderate or severe anxiety [19].

### Exposures

The intervention group participants were members of the women’s pelvic health program, which included access to a pelvic health exercise therapy plan, educational articles, and communication with a physical therapist and health coach. Throughout the course of the study, nonparticipants were emailed five of the clinical educational articles on pelvic health and pain management that participants in the intervention group received, but did not have access to the digital pelvic health program.

### Confounders

Model covariates included age, general health (poor, fair, good, very good, excellent), employment status (working, not working), duration of pain (less than 1 year, 1–2 years, more than 3 years), baseline pain, baseline anxiety, baseline depression, and the use of conservative and non-conservative healthcare services at baseline (no, yes). Conservative healthcare services included appointments with a doctor, physical therapist, orthopedic surgeon, or imaging, and non-conservative healthcare services included overnight stay in a hospital, emergency room visit, injections, or surgery.

### Data sources

All baseline data were collected from nonparticipants during study registration via an online survey. For intervention group participants, age and baseline pain were collected from the digital pelvic health program’s applicant questionnaire, and all other baseline data were collected from an online survey distributed at study registration. Additional data were collected from all participants via emailed surveys during the start of week 4 and during the start of week 12. Participants received up to three email reminders to complete the surveys. Upon completion of emailed surveys, both nonparticipant and intervention group respondents received gift cards for $10 at baseline, $10 at 4 weeks, and $20 at 12 weeks.

### Study size

Sample size calculations were based on the primary outcome of pain. Literature recommends a change of 10 points on the NPRS as the minimal clinically important difference in women with endometriosis-associated pelvic pain [20]. Assuming a more conservative, smaller-sized effect of 6.5 points (Cohen’s d = 0.33), standard deviation of 20.0 based on internal studies, 5% level of significance and 80% statistical power, the final analytic sample size estimate required 292 total participants, or 146 per group. Allowing for 45% attrition, the study aimed to recruit at least 648 participants, or 324 per group. Upon observing a higher attrition rate in the intervention group during recruitment, the sample size estimate for the intervention group was increased from 324 to 495 in order to ensure sample size estimates were met.

### Statistical methods

Summary statistics were calculated for age, pain the last 24 hours, general health, race and ethnicity, marriage status, employment status, education, and use of conservative or non-conservative healthcare. Two-tailed t-tests and chi-square tests were conducted to show whether there were significant differences between groups at baseline for continuous and categorical variables, respectively. Descriptive statistics were reported at 4 and 12 weeks for pain in the last 24 hours, moderate to severe anxiety, and moderate to severe depression.

Unadjusted and adjusted linear mixed effects regression models were conducted to model changes in pelvic pain, anxiety, and depression over time. Covariates were baseline age, duration of pain, anxiety, depression, overall health status, employment status, conservative healthcare use (appointments with a doctor, physical therapist, or orthopedic surgeon, or imaging), and non-conservative healthcare use (overnight stay in a hospital, emergency room visit, injections, or surgery). Time was treated as a categorical predictor in order to allow for the modeling of non-linear changes over time. A two-way time and group interaction captured the treatment effect at each time point. Estimated predicted probabilities and marginal effects are presented below.

The primary analysis used all available data. The maximum likelihood estimation was used, assuming data were missing at random. Analyses were performed using R statistical software (version 4.0.5; R Foundation for Statistical Computing).

## Results

### Flowchart

**Fig. 2:**
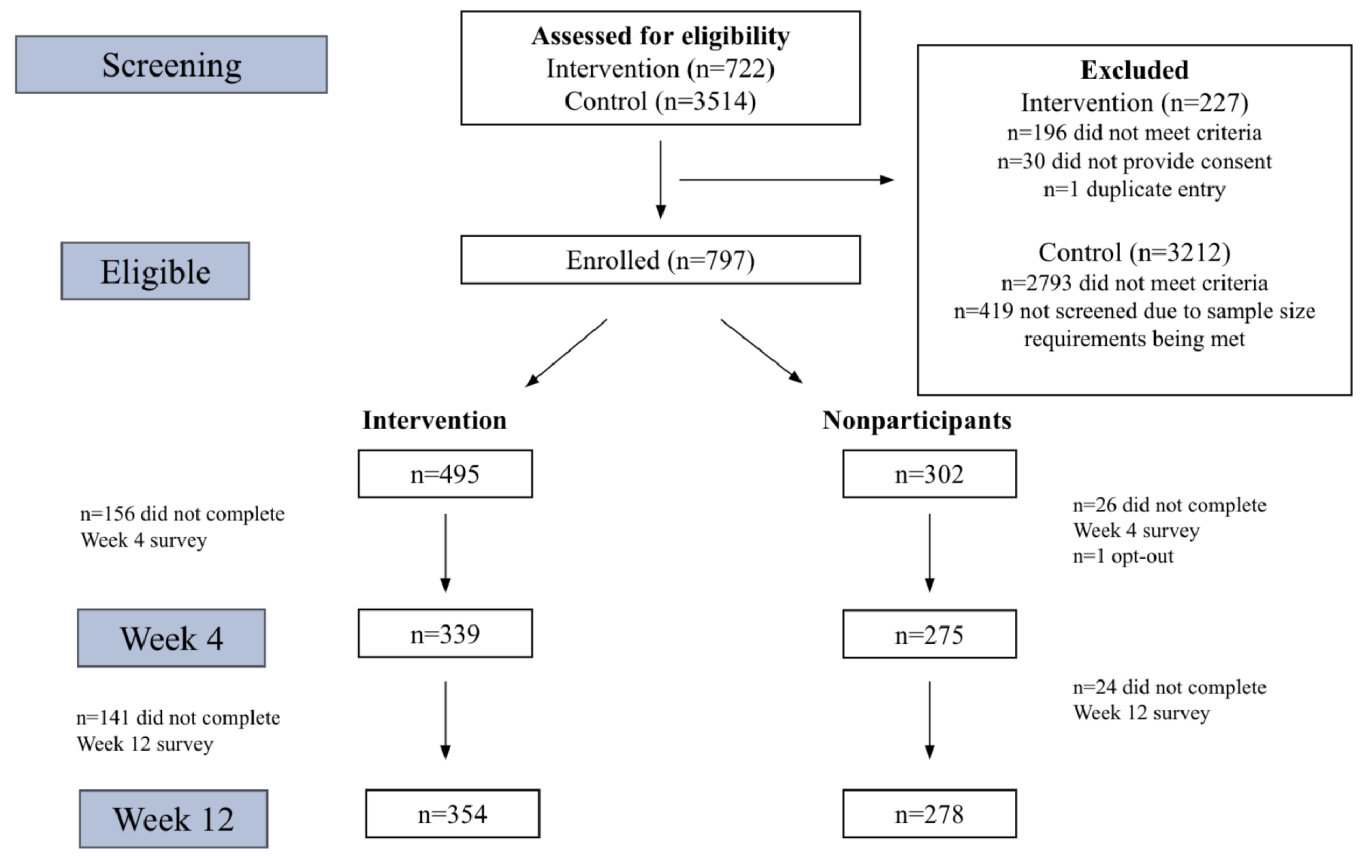
Flowchart, by group

Figure 2 reports the intervention and nonparticipant groups at each study stage. Overall, the response rates were 77.0% at 4 weeks (614/797) and 79.3% at 12 weeks (632/797).

### Sample characteristics

Table 1 presents the characteristics of sampled nonparticipant and intervention groups. Overall, 302 nonparticipants and 495 intervention group participants were included in the study. The mean (SD) age of the total sample was 41.5 (SD 11.7) years. At registration, the mean pain in the last 24 hours was 45.7 (18.5), out of 100. The majority of study participants were in good overall health (46.0%), white (62.0%), married (70.0%), and working (76.5%). All variables were significantly different between groups at baseline. The intervention group was younger, had lower pain, and had higher education levels than the nonparticipant group.

**Table 1:** Study sample characteristics at baseline

	Nonparticipants (*N* = 302)	Intervention (*N* = 495)	Total (*N* = 797)
Age*			
Mean (SD)	43.6 (14.4)	40.2 (9.45)	41.5 (11.7)
Median [Min, Max]	42.0 [18.0, 78.0]	38.0 [21.0, 73.0]	39.0 [18.0, 78.0]
Pain in the last 24 h*			
Mean (SD)	48.8 (19.9)	43.8 (17.3)	45.7 (18.5)
Median [Min, Max]	50.0 [20.0, 100]	42.0 [20.0, 100]	45.0 [20.0, 100]
General Health*			
Poor	4 (1.3%)	6 (1.2%)	10 (1.3%)
Fair	66 (21.9%)	62 (12.5%)	128 (16.1%)
Good	140 (46.4%)	227 (45.9%)	367 (46.0%)
Very good	74 (24.5%)	147 (29.7%)	221 (27.7%)
Excellent	15 (5.0%)	35 (7.1%)	50 (6.3%)
Race & Ethnicity*			
White	159 (52.6%)	335 (67.7%)	494 (62.0%)
Black or African American	78 (25.8%)	50 (10.1%)	128 (16.1%)
Asian	9 (3.0%)	24 (4.8%)	33 (4.1%)
Other Single and Multiple Races	54 (17.9%)	69 (13.9%)	123 (15.4%)
Marriage Status*			
Married or Living with Partner	170 (56.3%)	388 (78.4%)	558 (70.0%)
Widowed, Divorced, Separated, or Never Married	130 (43.0%)	91 (18.4%)	221 (27.7%)
Employment Status*			
Working (for pay, not for pay)	183 (60.6%)	427 (86.3%)	610 (76.5%)
Not working, Student, Retired, or Other	117 (38.7%)	50 (10.1%)	167 (21.0%)
Education*			
Less than High School, High School, Some College or Associate Degree	165 (54.6%)	155 (31.3%)	320 (40.2%)
Bachelor, Master, or Doctorate Degree	135 (44.7%)	324 (65.5%)	459 (57.6%)
Conservative healthcare use* **(appointments with a doctor**,** physical therapist**,** orthopedic surgeon**,** or imaging)**			
No	106 (35.1%)	217 (43.8%)	323 (40.5%)
Yes	191 (63.2%)	168 (33.9%)	359 (45.0%)
Non-conservative healthcare use* **(overnight stay in a hospital**,** emergency room visit**,** injections**,** or surgery)**			
No	106 (35.1%)	365 (73.7%)	471 (59.1%)
Yes	48 (15.9%)	24 (4.8%)	72 (9.0%)
Pain Duration*			
Less than 1 year	68 (22.5%)	177 (35.8%)	245 (30.7%)
1–2 years	80 (26.5%)	113 (22.8%)	193 (24.2%)
More than 3 years	154 (51.0%)	188 (38.0%)	342 (42.9%)
Moderate/Severe Anxiety*			
No	159 (52.6%)	334 (67.5%)	493 (61.9%)
Yes	142 (47.0%)	154 (31.1%)	296 (37.1%)
Moderate/Severe Depression*			
No	199 (65.9%)	394 (79.6%)	593 (74.4%)
Yes	102 (33.8%)	94 (19.0%)	196 (24.6%)

* *p* < 0.05

### Descriptive results

Nonparticipants’ absolute decrease in pain from baseline was 8.4 points at 4 weeks and 10.0 at 12 weeks. The intervention group’s absolute decrease in pain from baseline was 19.0 at 4 weeks and 22.2 at 12 weeks (Table 2).

The percentage who screened positive for moderate or severe anxiety was higher for the nonparticipant group than the intervention group by 15.6% at baseline, 20.0% at 4 weeks, and 18.9% at 12 weeks. The percentage who screened positive for moderate or severe depression was higher for the nonparticipant group than the intervention group by 14.6% at baseline, 16.4% at 4 weeks, and 21.7% at 12 weeks (Table 2).

**Table 2:** Descriptive results of pain, anxiety, and depression over time for nonparticipant and intervention groups

	Nonparticipants	Intervention
**Pain, mean (SD)**		
Baseline	48.8 (19.9)	43.8 (17.3)
Week 4	40.4 (25.8)	24.8 (22.4)
Week 12	38.8 (25.2)	21.6 (22.5)
**Screened in for moderate/severe anxiety**,** % (n)**		
Baseline	47.2% (142/301)	31.6% (154/488)
Week 4	42.0% (115/274)	22.0% (73/332)
Week 12	37.1% (103/278)	18.2% (64/351)
**Screened in for moderate/severe depression**,** % (n)**		
Baseline	33.9% (102/301)	19.3% (94/488)
Week 4	28.5% (79/274)	12.1% (40/330)
Week 12	31.7% (88/278)	10.0% (35/351)

Engagement in the digital women’s health app was also measured. On average, intervention group participants completed 13.7 (SD 14.4; median 8.0; range 1–76) exercise therapy sessions and read 11.5 (SD 13.4; median 6.0; range: 0–58) educational articles in the first 12 weeks after onboarding.

### Main results

**Fig. 3:**
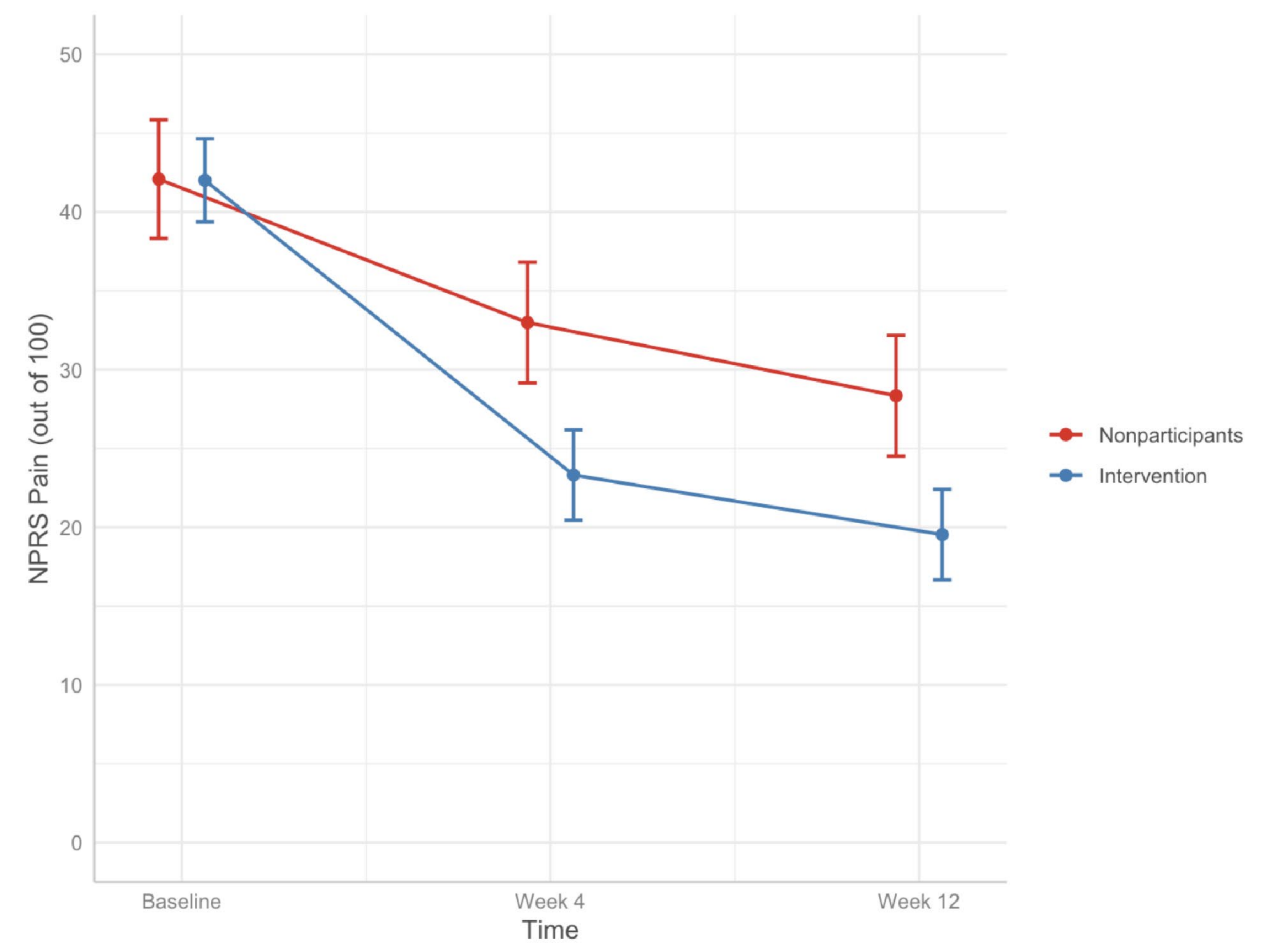
Adjusted NPRS scores over time. Results adjusted for age, general heath, employment status, duration of pain, baseline pain, baseline anxiety, baseline depression, healthcare service use, and time as fixed effects

The intervention group showed significantly lower adjusted pain scores at both follow-up timepoints compared to nonparticipants (Fig. 3). For nonparticipants, adjusted pain scores decreased from 42.1 (95% CI: [38.3, 45.9]) at baseline to 33.0 (95% CI: [29.2, 36.8]) at 4 weeks to 28.3 (95% CI: [24.5, 32.2]) at 12 weeks. For the intervention group, adjusted pain scores decreased from 42.0 (95% CI: [39.4, 44.7]) at baseline to 23.3 (95% CI: [20.5, 26.2]) at 4 weeks to 19.5 (95% CI: [16.7, 22.4]) at 12 weeks.

**Fig. 4:**
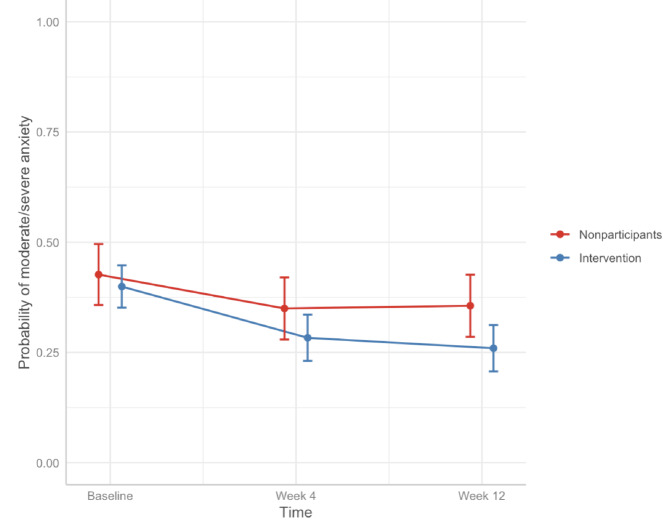
Adjusted probabilities of having moderate/severe anxiety over time. Results adjusted for age, general health, employment status, duration of pain, baseline pain, baseline anxiety, baseline depression, healthcare service use, and time as fixed effects.

There were no significant differences in anxiety outcomes between groups (Fig. 4). After adjustments, the probability of nonparticipants screening positive for moderate or severe anxiety was 0.43 (95% CI: [0.36, 0.50]) at baseline, 0.35 (95% CI: [0.28, 0.42]) at 4 weeks, and 0.36 (95% CI: [0.29, 0.43]) at 12 weeks. The adjusted probability of the intervention group screening positive for moderate or severe anxiety was 0.40 (95% CI: [0.35, 0.45]) at baseline, 0.28 (95% CI: [0.23, 0.34]) at 4 weeks, and 0.26 (95% CI: [0.21, 0.31]) at 12 weeks.

**Fig. 5:**
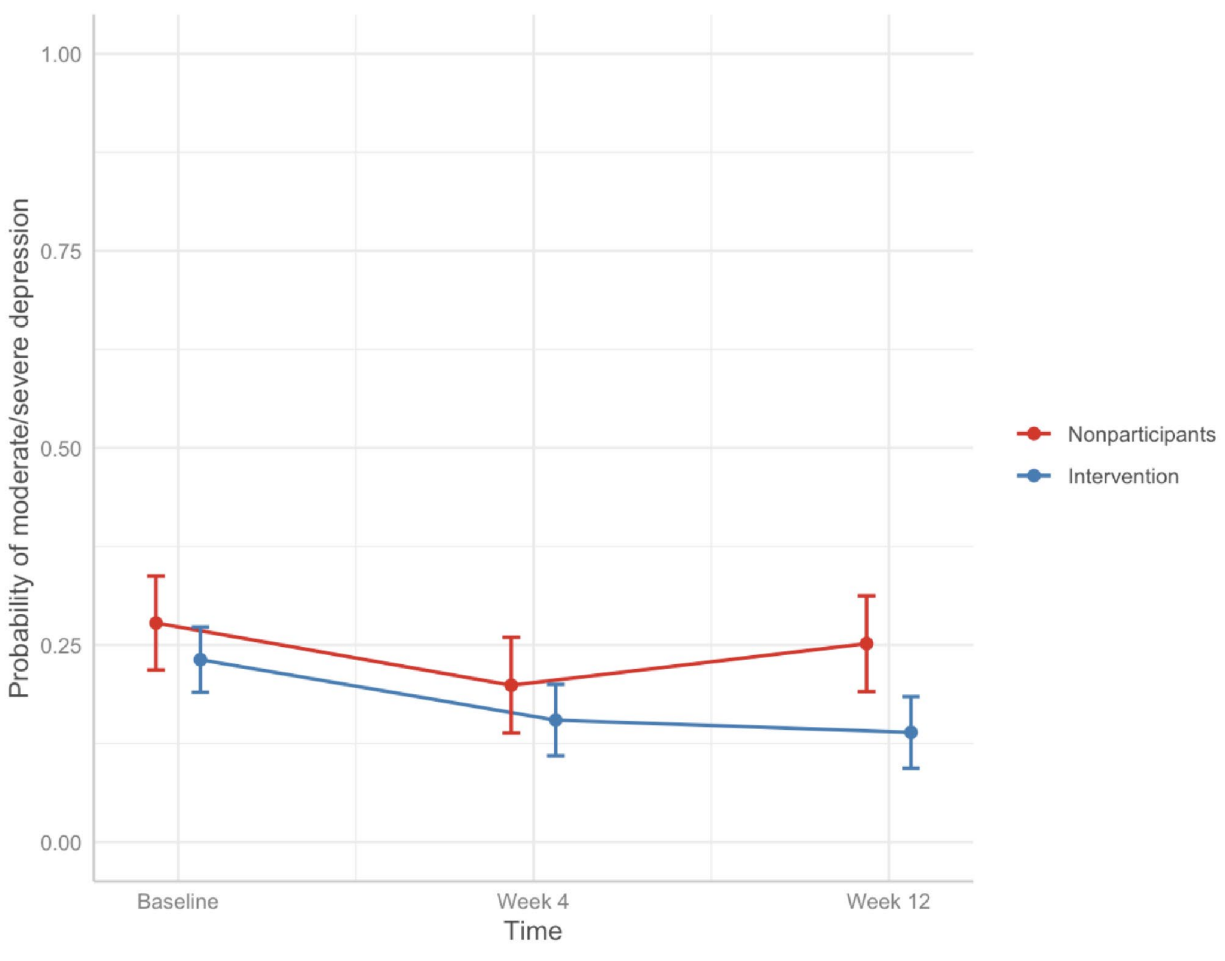
Adjusted probabilities of having moderate/severe depression over time. Results adjusted for age, general heatlh, employment status, duration of pain, baseline pain, baseline anxiety, baseline depression, healthcare service use, and time as fixed effects.

The intervention group showed a significantly lower probability of having moderate or severe depression at 12 weeks compared to nonparticipants (Fig. 5). The adjusted probability of nonparticipants having moderate or severe depression decreased from 0.28 (95% CI: [0.22, 0.34]) at baseline to 0.20 (95% CI: [0.14, 0.26]) at 4 weeks, and then increased to 0.25 (95% CI: [0.19, 0.31]) at 12 weeks. In comparison, the adjusted probability of the intervention group having moderate or severe depression decreased from 0.23 (95% CI: [0.19, 0.27]) at baseline to 0.15 (95% CI: [0.11, 0.20]) at 4 weeks to 0.14 (95% CI: [0.09, 0.18]) at 12 weeks.

Additional results, including both unadjusted and adjusted regression model results are included in Supplementary File 1.

## Discussion

This observational study examined pain, depression and anxiety outcomes at 4 and 12 weeks among participants of a digital women’s pelvic health program compared to a nonparticipant group. We found significant differences in pain between the intervention and nonparticipant groups at 4 and 12 weeks. Additionally, the study showed that participants of a digital women’s pelvic health program had a significantly lower probability of having moderate or severe depression at 12 weeks.

Based on adjusted results, the percentage of pain improvement from baseline was higher for the intervention versus the nonparticipant group by 22.9% at 4 weeks and 20.9% at 12 weeks. While there is little research published on the effectiveness of digital women’s pelvic health programs on pelvic pain reduction, the research that exists suggests digital therapeutics can be highly effective. A randomized controlled trial studying a digital therapeutic treatment for women with endometriosis found the average pain relief to be significantly higher in the intervention group at 28% compared to a control group at 15% [21]. Additionally, a pilot randomized trial comparing a virtual reality exercise program and telehealth-delivered exercise program to a control group found that both digital therapeutic interventions provided immediate relief from endometriosis-associated pelvic pain after a single session [22].

While this study found that participants in a digital women’s pelvic health program had a significantly lower probability of having moderate or severe depression than nonparticipants at 12 weeks, no significant difference in anxiety was found between groups. Research suggests that anxiety and depression are often maintained by pain-related fear [23], however, evidence-based ways to improve anxiety and depression for this population are understudied. One reason no significant differences in anxiety were observed may be because the GAD-2 screener specifically measures generalized anxiety and may not have captured the anxiety or fear avoidance that is more relevant to pelvic disorders. As mental health has a major impact on pelvic floor treatment adherence, engagement, and treatment effectiveness, additional research on mental health and fear avoidance outcomes is needed.

Both strengths and limitations of this study should be considered when interpreting its results. To our knowledge, this is the first study that includes a comparison group to examine the efficacy of a digital women’s pelvic health program on decreasing pelvic pain. In order to properly demonstrate the effects of the digital pelvic health program, a comparison group was included. Furthermore, multiple clinical outcomes were measured in both the short and medium term, and the digital pelvic health program was evaluated in real-world settings. Findings of this study are generalizable to a population with chronic pelvic pain with expressed interest in a digital women’s pelvic health program. This study also presents its limitations. As an observational study, findings from this study cannot establish the causality of the intervention’s effect on outcomes. Given that the nonparticipant group was recruited through a recruitment firm, nonparticipants were likely more interested in participating in the study and had higher response rates compared to intervention participants, suggesting possible attrition bias. The differences in recruitment methods may have also introduced treatment selection bias, given the between-group differences in baseline characteristics between groups. To address this concern, we ensured that these differences were controlled for in adjusted analyses and reported both the unadjusted and adjusted results. We were unable to account for other influential factors that may have affected results in our analyses, such as lifestyle and amount of time spent exercising at baseline. Lastly, a validated clinical scale used to measure fear avoidance might have been better suited for measuring fear and anxiety related to pelvic pain.

To address these limitations, future research could include prospectively designed randomized controlled trials to establish stronger validity and generalizability. While this study examined clinical outcomes at both 4 and 12 weeks, longer follow-up would increase the understanding of the sustained effects of the digital women’s health program. Further examination into program engagement and how it may correlate to outcomes should be considered in future analyses. Lastly, a component analysis is recommended to determine which aspects of the program are most effective at reducing pelvic pain and improving quality of life.

## Conclusion

Results of this study suggest a digital women’s pelvic health program focused on chronic pelvic pain management is feasible and effective at reducing pelvic pain and depression in the short and medium term. This study contributes to the growing body of evidence on the efficacy of telehealth therapies designed to treat women’s pelvic pain, and highlights the necessity for expanded research on the interaction between mental health and pelvic pain.

## Electronic supplementary material

Below is the link to the electronic supplementary material.

Supplementary Material 1

## Data Availability

De-identified data are available from the corresponding author upon reasonable request.

## Abbreviations

CPP: Chronic Pelvic Pain
PFPT: Pelvic floor physical therapy (PFPT)
NPRS: Numeric Pain Rating Scale
PT: Physical Therapy
PHQ-2: Patient Health Questionnaire 2-Item
GAD-2: Generalized Anxiety Disorder 2-Item
SD: Standard Deviation
CI: Confidence Interval
