# Supplementary material for: Clinical outcomes of a digital musculoskeletal women’s pelvic health program: an observational, longitudinal study with comparison group
Source: BMC Womens Health. 2025 Jan 11;25:18. doi: 10.1186/s12905-024-03475-4 (PMC11724504; doi:10.1186/s12905-024-03475-4)
Supplement: Supplementary file 1 — Supplementary Material 1 [file 12905_2024_3475_MOESM1_ESM.docx]

# **Supplementary Files**

Clinical outcomes of a digital musculoskeletal women’s pelvic health program: an observational, longitudinal study with comparison group

**Supplementary File 1.** Unadjusted values and probabilities of primary and secondary outcomes for intervention and nonparticipant groups

**Pain**

**Figure S1.** Unadjusted NPRS scores over time.


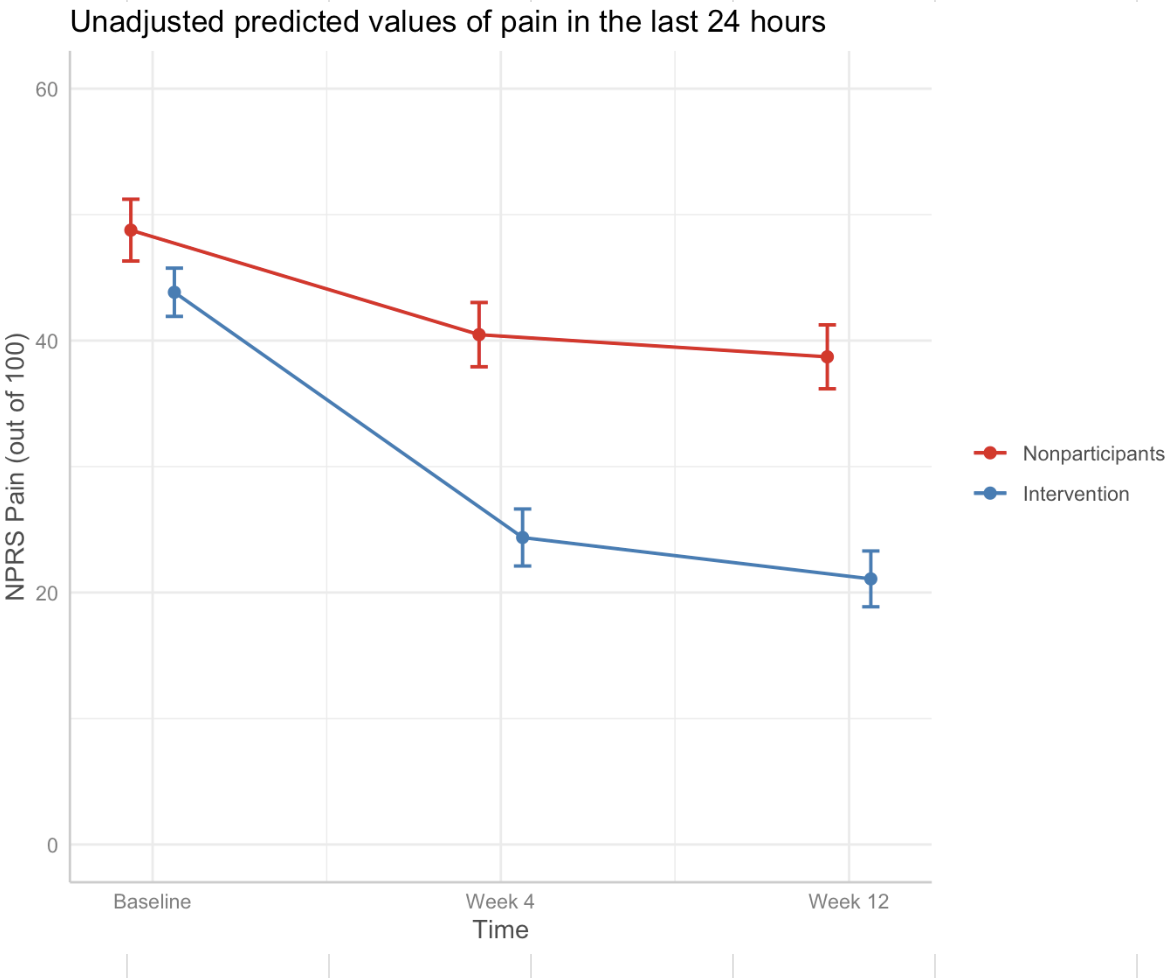


**Table S1.** Unadjusted values of pain in the last 24 hours

| **Non-participants** | **Estimate** | **95% CI (lower)** | **95% CI (upper)** |
| --- | --- | --- | --- |
| Baseline | 48.77 | 46.32 | 51.22 |
| Week 4 | 40.47 | 37.92 | 43.02 |
| Week 12 | 38.71 | 36.17 | 41.25 |
| **Intervention** |  |  |  |
| Baseline | 43.84 | 41.92 | 45.75 |
| Week 4 | 24.37 | 22.11 | 26.63 |
| Week 12 | 21.08 | 18.87 | 23.30 |

**Anxiety**

**Figure S2.** Unadjusted probabilities of having moderate/severe anxiety over time.


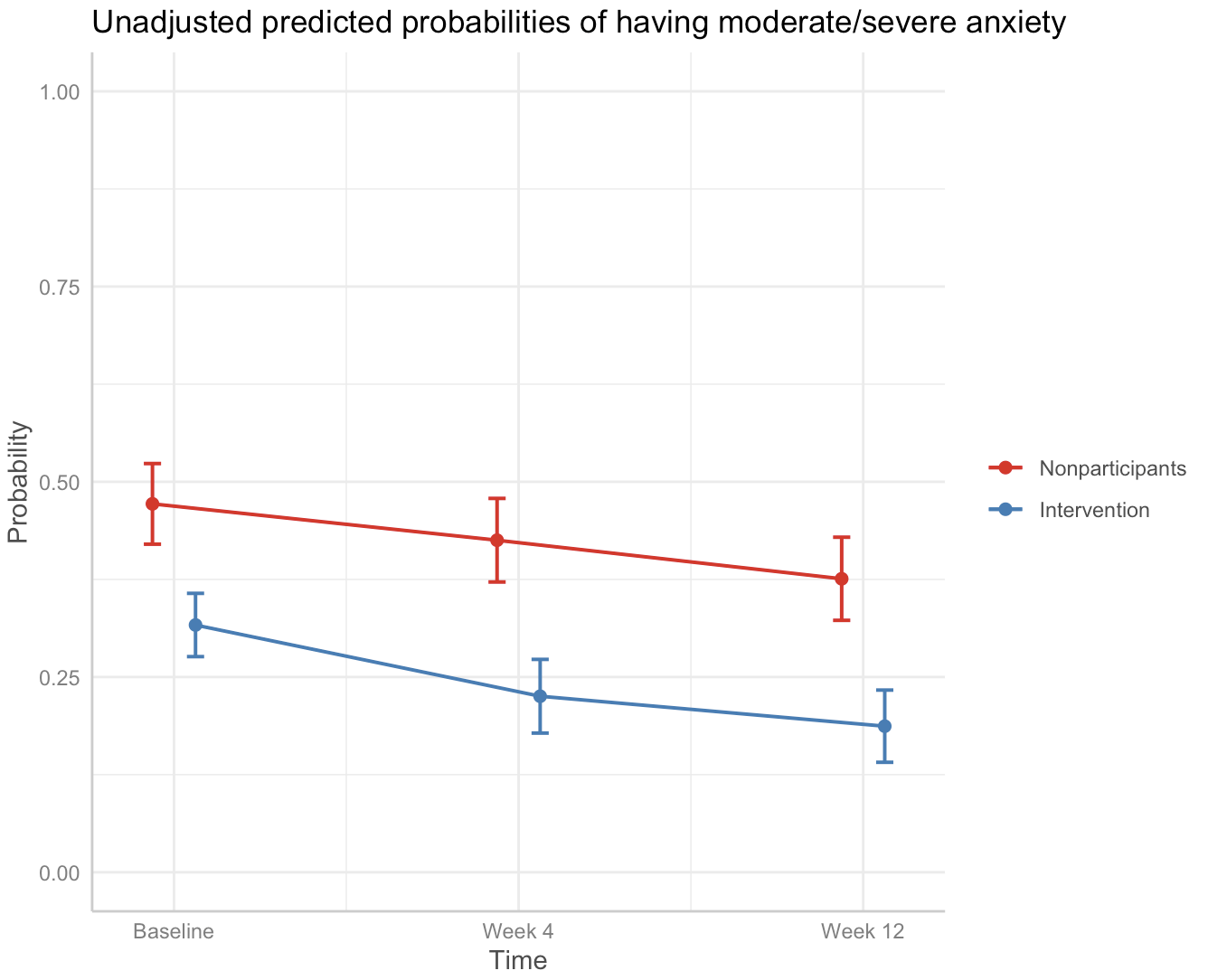


**Table S2.** Unadjusted probabilities of having moderate/severe anxiety

| **Non-participants** | **Estimate** | **95% CI (lower)** | **95% CI (upper)** |
| --- | --- | --- | --- |
| Baseline | 0.47 | 0.42 | 0.52 |
| Week 4 | 0.43 | 0.37 | 0.48 |
| Week 12 | 0.38 | 0.32 | 0.43 |
| **Intervention** |  |  |  |
| Baseline | 0.32 | 0.28 | 0.36 |
| Week 4 | 0.23 | 0.18 | 0.27 |
| Week 12 | 0.19 | 0.14 | 0.23 |

**Depression**

**Figure S3.** Unadjusted probabilities of having moderate/severe depression over time.

**
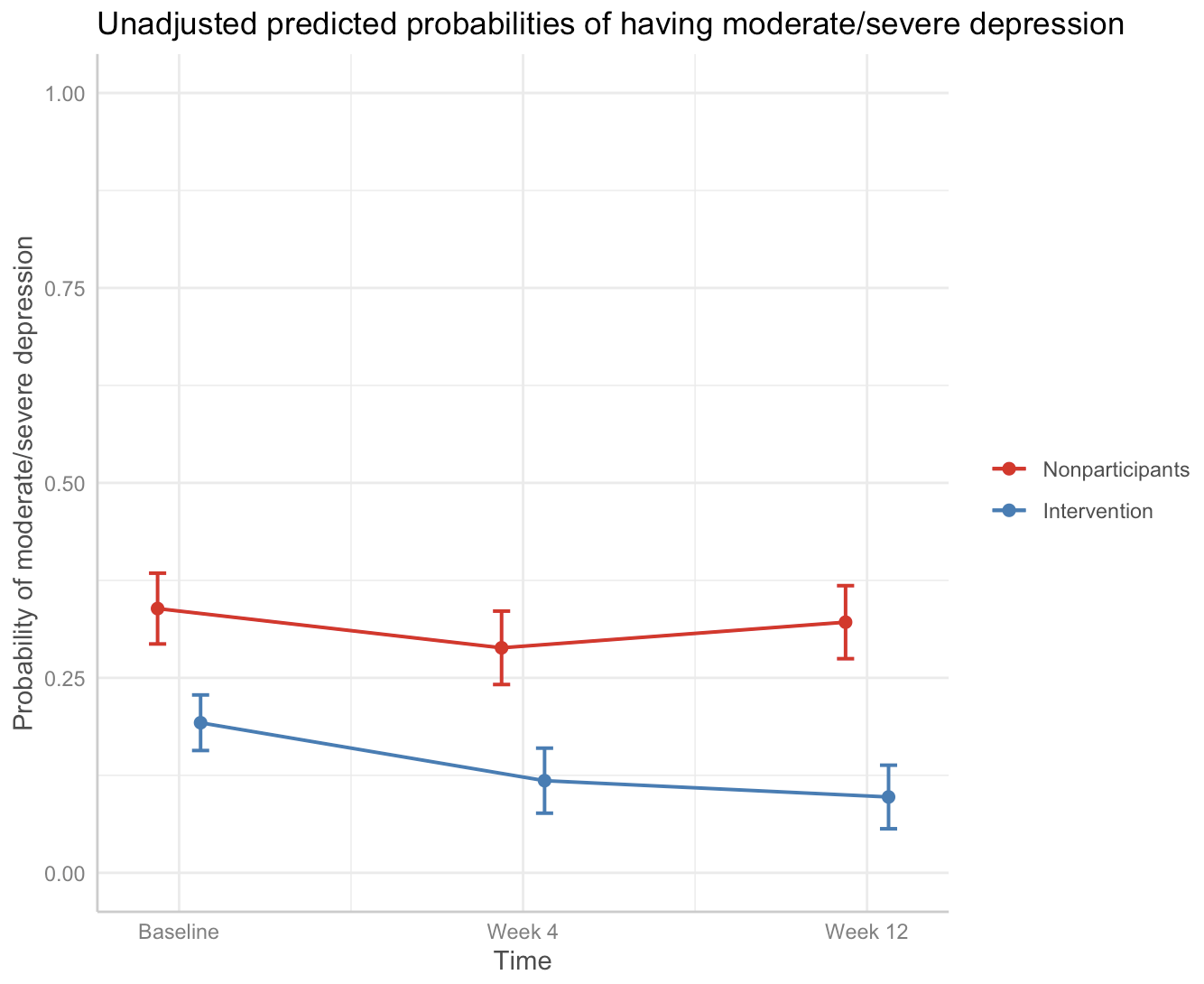
**

**Table S3.** Unadjusted probabilities of having moderate/severe depression

| **Non-participants** | Estimate | 95% CI (lower) | 95% CI (upper) |
| --- | --- | --- | --- |
| Baseline | 0.34 | 0.29 | 0.38 |
| Week 4 | 0.29 | 0.24 | 0.34 |
| Week 12 | 0.32 | 0.27 | 0.37 |
| **Intervention** |  |  |  |
| Baseline | 0.19 | 0.16 | 0.23 |
| Week 4 | 0.12 | 0.08 | 0.16 |
| Week 12 | 0.10 | 0.06 | 0.14 |

**Supplementary File 2.** Unadjusted and adjusted model results comparing the intervention group to nonparticipants for primary and secondary outcomes

**Table S4.** Unadjusted and adjusted model results for pain

|  | Unadjusted | | | Adjusted | | |
| --- | --- | --- | --- | --- | --- | --- |
|  | Estimate | 95% CI (lower) | 95% CI (upper) | Estimate | 95% CI (lower) | 95% CI (upper) |
| intervention | -4.93 | -8.04 | -1.82 | -0.07 | -3.89 | 3.74 |
|  |  |  |  |  |  |  |
| weeks |  |  |  |  |  |  |
| 4 | -8.30 | -11.08 | -5.52 | -9.10 | -12.93 | -5.28 |
| 12 | -10.06 | -12.83 | -7.29 | -13.74 | -17.55 | -9.92 |
|  |  |  |  |  |  |  |
| intervention*weeks |  |  |  |  |  |  |
| intervention * 4 | -11.17 | -14.86 | -7.48 | -9.60 | -14.22 | -4.95 |
| intervention * 12 | -12.69 | -16.34 | -9.05 | -8.74 | -13.36 | -4.11 |
|  |  |  |  |  |  |  |
|  |  |  |  | Results adjusted for age, general heath, employment status, duration of pain, baseline pain, baseline anxiety, baseline depression, healthcare service use, and time as fixed effects. Intervention*weeks indicates the interaction term between the two variables. | | |

**Table S5.** Unadjusted and adjusted model results for anxiety

|  | Unadjusted | | | Adjusted | | |
| --- | --- | --- | --- | --- | --- | --- |
|  | Odds Ratio | 95% CI (lower) | 95% CI (upper) | Odds Ratio | 95% CI (lower) | 95% CI (upper) |
| intervention | 0.86 | 0.80 | 0.91 | 0.96 | 0.90 | 1.02 |
|  |  |  |  |  |  |  |
| weeks |  |  |  |  |  |  |
| 4 | 0.95 | 0.90 | 1.01 | 0.92 | 0.87 | 0.99 |
| 12 | 0.98 | 0.86 | 0.96 | 0.97 | 0.91 | 1.04 |
|  |  |  |  |  |  |  |
| intervention*weeks |  |  |  |  |  |  |
| intervention * 4 | 0.98 | 0.89 | 1.03 | 1.00 | 0.93 | 1.09 |
| intervention * 12 | 0.93 | 0.90 | 1.04 | 0.94 | 0.86 | 1.01 |
|  |  |  |  |  |  |  |
|  |  |  |  | Results adjusted for age, general heath, employment status, duration of pain, baseline pain, baseline anxiety, baseline depression, healthcare service use, and time as fixed effects. Intervention*weeks indicates the interaction term between the two variables. | | |

**Table S6.** Unadjusted and adjusted model results for depression

|  | Unadjusted | | | Adjusted | | |
| --- | --- | --- | --- | --- | --- | --- |
|  | Odds Ratio | 95% CI (lower) | 95% CI (upper) | Odds Ratio | 95% CI (lower) | 95% CI (upper) |
| intervention | 0.86 | 0.80 | 0.91 | 0.96 | 0.90 | 1.02 |
|  |  |  |  |  |  |  |
| weeks |  |  |  |  |  |  |
| 4 | 0.95 | 0.90 | 1.01 | 0.92 | 0.87 | 0.99 |
| 12 | 0.98 | 0.86 | 0.96 | 0.97 | 0.91 | 1.04 |
|  |  |  |  |  |  |  |
| intervention*weeks |  |  |  |  |  |  |
| intervention * 4 | 0.98 | 0.89 | 1.03 | 1.00 | 0.93 | 1.09 |
| intervention * 12 | 0.93 | 0.90 | 1.04 | 0.94 | 0.86 | 1.01 |
|  |  |  |  |  |  |  |
|  |  |  |  | Results adjusted for age, general heath, employment status, duration of pain, baseline pain, baseline anxiety, baseline depression, healthcare service use, and time as fixed effects. Intervention*weeks indicates the interaction term between the two variables. | | |
